# Perivascular Spaces Segmentation in Brain MRI Using Optimal 3D Filtering

**DOI:** 10.1038/s41598-018-19781-5

**Published:** 2018-02-01

**Authors:** Lucia Ballerini, Ruggiero Lovreglio, Maria del C. Valdés Hernández, Joel Ramirez, Bradley J. MacIntosh, Sandra E. Black, Joanna M. Wardlaw

**Affiliations:** 1 Department of Neuroimaging Sciences, Centre for Clinical Brain Sciences, Centre for Cognitive Ageing and Cognitive Epidemiology and UK Dementia Research Institute Edinburgh Dementia Research Centre, University of Edinburgh, Edinburgh, UK; 2 Department of Civil and Environmental Engineering, University of Auckland, Auckland, New Zealand; 3 Hurvitz Brain Sciences Program, LC Campbell Cognitive Neurology Research Unit, Heart and Stroke Foundation Canadian Partnership for Stroke Recovery, Sunnybrook Research Institute and the University of Toronto, Toronto, Ontario Canada

## Abstract

Perivascular Spaces (PVS) are a feature of Small Vessel Disease (SVD), and are an important part of the brain’s circulation and glymphatic drainage system. Quantitative analysis of PVS on Magnetic Resonance Images (MRI) is important for understanding their relationship with neurological diseases. In this work, we propose a segmentation technique based on the 3D Frangi filtering for extraction of PVS from MRI. We used ordered logit models and visual rating scales as alternative ground truth for Frangi filter parameter optimization and evaluation. We optimized and validated our proposed models on two independent cohorts, a dementia sample (N = 20) and patients who previously had mild to moderate stroke (N = 48). Results demonstrate the robustness and generalisability of our segmentation method. Segmentation-based PVS burden estimates correlated well with neuroradiological assessments (Spearman’s *ρ* = 0.74, p < 0.001), supporting the potential of our proposed method.

## Introduction

**Figure 1:**
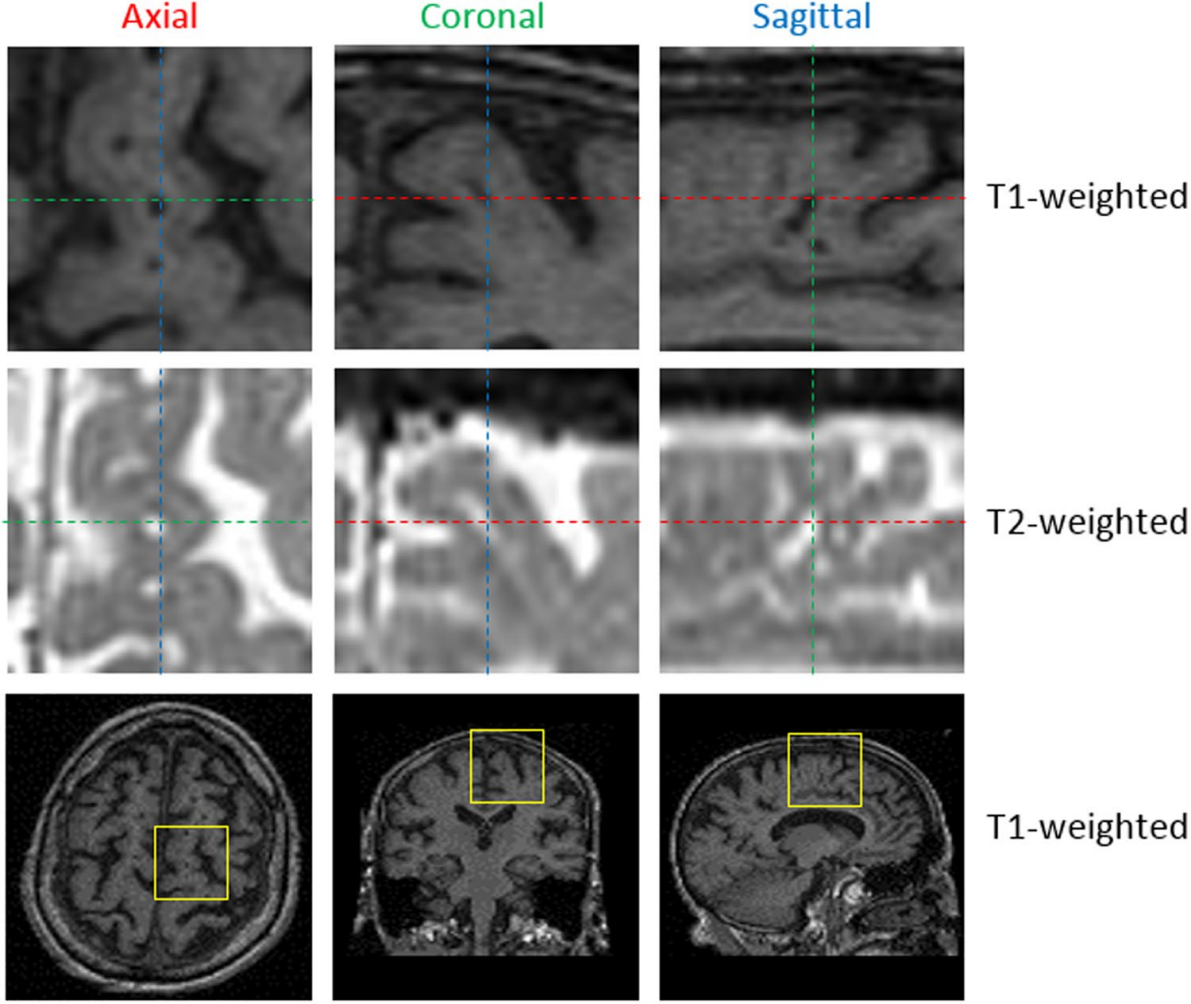
Magnified view of PVS in an axial, coronal and sagittal slice in T1-weighted and T2-weighted MR images. The position of these zooms in corresponding T1-weighted brain scans is highlighted with yellow squares (bottom row).

Perivascular spaces (PVS), also known as Virchow-Robin spaces, are fluid-filled spaces that follow the typical course of cerebral penetrating vessels. PVS have the same Magnetic Resonance Imaging (MRI) contrast characteristics as Cerebrospinal Fluid (CSF), that is they appear hypointense (dark) on T1-weighted (T1) and hyperintense (bright) on T2-weighted images (T2)^1,2^. They appear as small 3D tubular structures that, depending on the viewing plane, are linear or round, with a diameter generally smaller than 3 mm^3^ (see Fig. 1)

.

Enlargement of perivascular spaces is associated with other morphological features of Small Vessel Disease (SVD) such as white matter hyperintensities and lacunes^4^; cognitive impairment^5^ and inflammation^6^. Most studies use visual rating scales to assess PVS burden^7,8^, but these are prone to inter-observer variability, particularly in the Centrum Semiovale, due to the coexistence of PVS with other neuroradiological features of SVD that confound their identification in this region^7^.

Efforts have been made to computationally assess PVS^3,9^. Recent semi-automatic methods are based on thresholding and require user intervention either for the choice of parameters or for manual editing of the resulting masks, which, for small and frequent features such as PVS, risks introducing inter-observer variability and is very time consuming^10,11^. A promising approach proposed for PVS automatic segmentation uses the Frangi filter^12^ parameterised through a Random Forest scheme^13^ that learns discriminative PVS characteristics from manually segmented ground truth on MR images acquired at 7 T^14–16^. However, MRI in clinical research and practice is mostly performed in scanners with field strengths at 1.5 T or 3 T, and the reference standards available are visual ratings performed by neuroradiologists, which restricts the learning-based approach proposed by Park *et al*.^15^ in practice. Moreover, it is difficult to assess enlarged PVS burden at high field 7 T MRI since normal PVS and deep medullary veins with similar intensities to PVS confound visualization and requires observer correction.

Our current goal is to present a segmentation approach for enlarged PVS that can be used widely in current clinical research studies, to further elucidate their pathological significance and assess their potential role in neurological disorders. The main innovation of this paper is a method for optimization and evaluation of the filter in absence of ground truth segmentation. In other words, this method allows to use labels requiring little annotation effort to derive much finer results (pixel-wise segmentation).

We propose a novel application of ordered logit models, usually used in statistics as a regression model for ordinal dependent variables, as this model provides a good estimate for capturing the sources of influence that explain the ordinal dependent variables (i.e. in this case the PVS visual rating scores) considering the uncertainty (i.e. subjectivity, inter-observer variability) in the measurement of such data^17^. We use this model to estimate the parameters of the Frangi filter^12^ to obtain the maximum likelihood of a vessel-like structure to be a PVS in the Centrum Semiovale, by also estimating the count of PVS that most likely falls in the class corresponding to the category given by the neuroradiologist in this brain region.

We calibrated different ordered logit models, according to the rating scale available for every dataset. We optimized the parameters of the Frangi filter to deal with T1-weighted (T1W) and T2-weighted (T2W) modalities, and combined the resulting filtered images. Validation was carried out on different cohorts, using images acquired in 2 different sites, rated by 3 different raters.

## Materials

Two datasets were used for developing, testing and validating the method:

1. *Sunnybrook Dementia Study* (SDS): a large registered ongoing longitudinal clinical trial conducted at Sunnybrook Health Science Centre, Toronto, Canada (ClinicalTrials.gov NCT01800214). The study has been approved by the Sunnybrook Research Ethics Board in accordance with the principles expressed in the Declaration of Helsinki. Each patient provided informed consent. Patients had an historical profile typical of Alzheimer’s disease (AD). Full study details have been published previously^10^.
2. *Mild Stroke Study* (MSS): a study conducted at Centre for Clinical Brain Science, Edinburgh, UK. Patients had clinical features of lacunar or mild cortical stroke. All experimental protocols were approved by the Lothian Ethics of Medical Research Committee (REC 09/81101/54) and the NHS Lothian R + D Office (2009/W/NEU/14) and conducted according to the principles expressed in the Declaration of Helsinki. All patients gave written informed consent. The MRI protocol has been published elsewhere^18^.

The characteristics of the sequences relevant for PVS assessment are summarized in Table 1.

**Table 1:** Characteristics of the relevant MRI sequences of (1) Sunnybrook Dementia Study (SDS)^10^, and (2) Mild Stroke Study (MSS)^18^.

Study/parameters		Matrix	Voxel size (*mm*^3^)	TE (*ms*)	TR (*ms*)	flip angle	FOV (*cm*)	bandwidth (*KHz*)	Acq. time (*min*)
**SDS**	T1	256 × 256 × 124	0.86 × 0.86 × 1.4	5	35	35°	22	31.26	11
	T2/PD^*^	256 × 256 × 58	0.78 × 0.78 × 3	30/80	3000	90°	20	11.36	12
**MSS**	T1	256 × 216 × 256	1.02 × 0.9 × 1.02	14	400	—	24	15.63	0.54
	T2	384 × 224 × 28	0.47 × 0.47 × 6	90	6000	—	24	50	2.30
	T2cube	512 × 512 × 256	0.47 × 0.47 × 0.7	143	3000	—	24	31.25	3.55

^*^PD = proton density (interleaved), available in SDS.

## Methods

**Figure 2:**
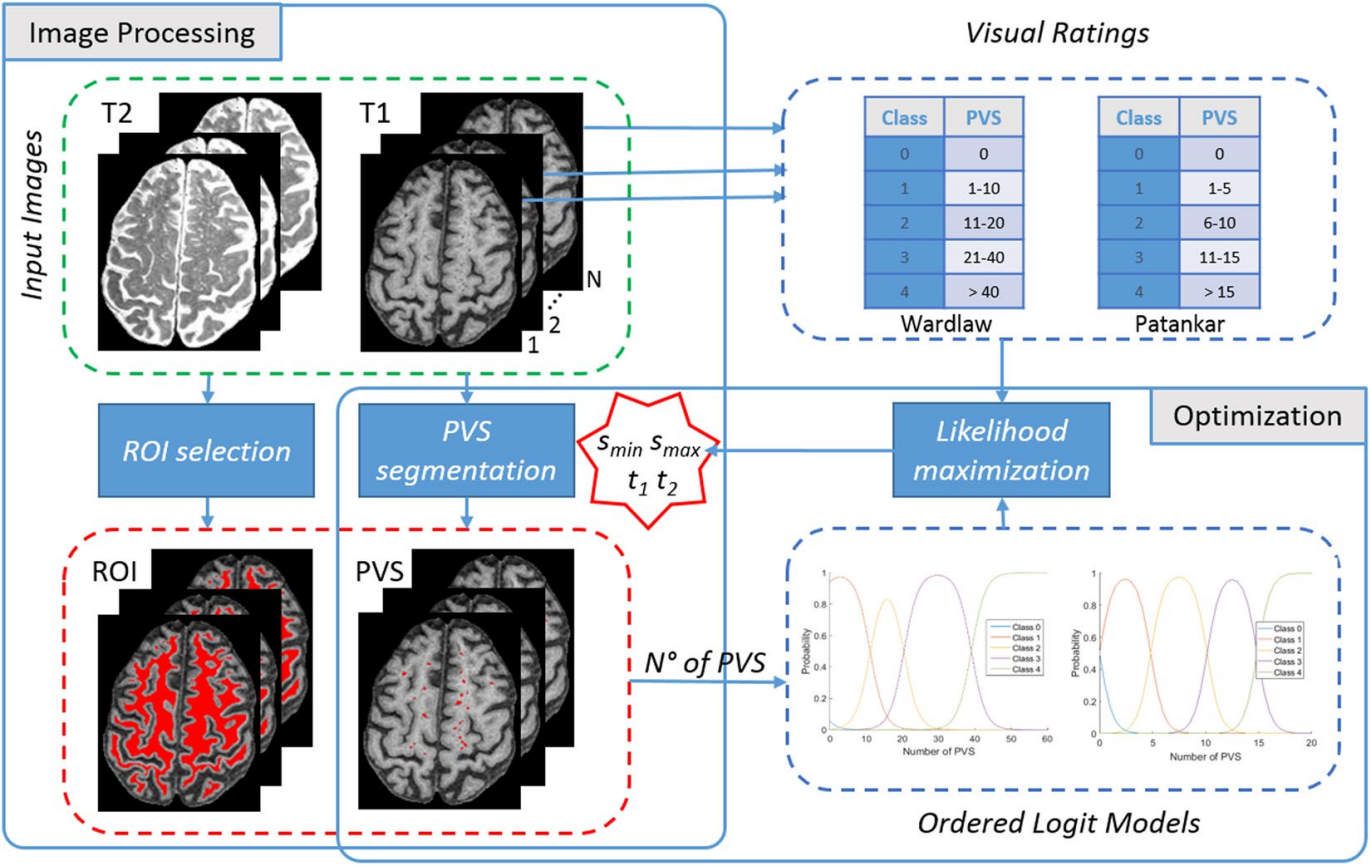
Framework of the proposed optimization approach: Frangi filter parameters (*s*_*min*_, *s*_*max*_) and thresholds (*t*_1_, *t*_2_) are optimized with order logit models and visual rating scales.

Observing the vessel-like structure of PVS, we propose a segmentation technique based on the 3D Frangi filtering^12^, largely used for enhancing blood vessels, for instance in retinal images^19^. Given the absence of an accurate computational “ground truth” (i.e. manual labels of each PVS by experts), we propose a modelling technique to use the available information (i.e. PVS burden assessed using visual rating scales) to optimize the filter parameters. For this scope, an ordered logit model^17^ has been used to simulate the relationship between the number of PVS and the rating categories, taking into account the uncertainty in the measurements. The framework of the proposed optimization process is illustrated in Fig. 2.

### PVS assessment

PVS masks, obtained as described in Ramirez *et al*.^10^, were available for the SDS dataset. These masks obtained using Lesion Explorer^20^, which implements 2 false positive minimization strategies: (i) in order to reduce errors from minor imaging artifacts and improve differentiation from lacunar infarcts, candidate PVS are required to satisfy acceptance criteria from both T1W and T2W, and rejection criteria from PD, and (ii) to address potential registration errors and partial volume effects, the cortical Gray Matter segmentation was dilated by 1 voxel. This resulted in a relatively conservative estimate of the overall PVS burden and thus, limited its utility as a Ground Truth (GT) for segmentation optimization, as well as for pixel-wise evaluation of the results.

Two established visual rating scales for PVS severity were used in the present work. Previous work has demonstrated their comparability^7,10^.

The visual rating scale developed by Potter *et al*.^7^ (in the following called Wardlaw scale) required users to rate PVS burden on T2-weighted MRI in each of three major anatomical brain regions: midbrain, basal ganglia and centrum semiovale. According to the online user guide (http://www.sbirc.ed.ac.uk/documents/epvs-rating-scale-user-guide.pdf), PVS in the latter region should be assessed in the slice and hemisphere with the highest number, and rated as 0 (no PVS), 1 (mild; 1–10 PVS), 2 (moderate; 11–20 PVS), 3 (frequent; 21–40 PVS) or 4 (severe; >40 PVS).

The PVS scores proposed by Patankar *et al*.^21^ were based principally on the appearances seen on T1W inversion recovery images. PVS should be scored in the centrum semiovale as 0 (none), 1 (less than five per side), 2 (more than five on one or both sides), reflecting the lesser visibility of PVS on T1W.

Two slightly modified versions of these rating methods, as previously described^10^, were also used in this work. Coregistered MRIs were used for assessment, with T2W for primary identification, T1W for confirmation, and Proton Density (PD) for rejection as required. To reduce ceiling effects and account for a greater range of PVS, the Patankar scale was standardized: 0 (none), 1 (one to five), 2 (six to ten), 3 (eleven to fifteen), 4 (sixteen or more). To reduce double-counting, a slice increment of 3 was implemented as a standardized rating protocol. Centrum Semiovale was defined as the White Matter (WM) projections superior to the ventricles, present in each of the cerebral hemispheres under the cerebral cortex.

PVS were assessed in 20 representative cases of the SDS dataset by three raters: two experienced neuroradiologists using the two modified Wardlaw and Patankar visual rating scales, and a third rater strictly following the guideline of the original Wardlaw^7^ and Patankar^21^ rating methods. The two ratings (modified Wardlaw and Patankar) of the first raters were close to the conservative estimate of PVS burden obtained as described above. Inter-rater reliability was high (ICC = 0.99, <0.001) as previously discussed^10^. The third rater counted all visible PVS in the slice with the highest number in T1W and T2W, including the very small ones discarded by the first raters. All raters were blind to each other.

### Frangi filter

Frangi^12^ analyses the second order derivatives of an image I, defined in the Hessian matrix *H*_*s*_(*v*) as:

1 $${H}_{s}(v)=[\begin{array}{ccc}{I}_{xx} & {I}_{xy} & {I}_{xz}\\ {I}_{yx} & {I}_{yy} & {I}_{yz}\\ {I}_{zx} & {I}_{zy} & {I}_{zz}\end{array}]$$

to describe the “vesselness” *F*(*v*) of a voxel *v* at scale *s* as:

2 $${F}_{s}(v)=\{\begin{array}{cc}0 & {\text{if}}\,{\lambda }_{2}\ge 0\\  & {\text{or}}\,{\lambda }_{3}\ge 0,\\ (1-{e}^{-\frac{{R}_{A}^{2}}{2{\alpha }^{2}}})\cdot {e}^{-\frac{{R}_{B}^{2}}{2{\beta }^{2}}}\cdot (1-{e}^{-\frac{{S}^{2}}{2{c}^{2}}}) & {\text{otherwise}},\end{array}$$

where *λ*_1_, *λ*_2_ and *λ*_3_ are the ordered eigenvalues (|*λ*_1_| ≤ |*λ*_2_| ≤ |*λ*_3_|) of the Hessian matrix, *R*_*A*_ = |*λ*_2_|/|*λ*_3_|, *R*_*B*_ = |*λ*_1_|/(|*λ*_2_*λ*_3_|)^1/2^, $S=({\lambda }_{1}^{2}+{\lambda }_{2}^{2}+{\lambda }_{3}^{2}{)}^{\mathrm{1/2}}$, and *α*, *β*, *c* are thresholds which control the sensitivity of the filter to the measures *R*_*A*_, *R*_*B*_ and *S*.

For a bright tubular structure in a 3D image we expect: |*λ*_1_| ≤ |*λ*_2_|, |*λ*_3_| and $|{\lambda }_{2}|\sim |{\lambda }_{3}|$; $|{\lambda }_{1}|\sim 0$ and *λ*_2_, *λ*_3_ ≤ 0. For a dark structure *λ*_2_, *λ*_3_ ≥ 0 and the conditions in Eq. 2 should be reversed.

Given a set of scales *s* ∈ [*s*_*min*_, *s*_*max*_], the responses are combined as:

3 $$F(v)=\mathop{{\rm{\max }}}\limits_{s}{F}_{s}(v)$$

where *s*_*min*_ and *s*_*max*_ are the minimum and maximum scales at which relevant structures are expected to be found^12^.

### Ordered Logit Model

An ordered logit model defines the relationship between an ordinal variable (*y*) which can vary between 0 and *m*(*m* ∈ *N*^+^), and the vector of independent variables (*x*) by using a latent continuous variable (${y}^{\ast }$) defined in an one-dimensional space characterized by threshold points (*μ*_0_, …, *μ*_*m*−1_) as described in equation:

4 $${y}^{\ast }=\beta x+\varepsilon ,\,\varepsilon  \sim G(\mu |\sigma ),\mu =0,\,\sigma =\pi /\sqrt{3}$$

5 $$\begin{array}{ccc}{y}_{i}=0 & if & -\infty  < {y}_{i}^{\ast }\le {\mu }_{0}\\ {y}_{i}=1 & if & {\mu }_{0} < {y}_{i}^{\ast }\le {\mu }_{1}\\ \,\cdots  &  & \\ {y}_{i}=m & if & {\mu }_{m-1} < {y}_{i}^{\ast }\le \infty \end{array}$$

where *β* and *μ*_*i*_ are parameters to be estimated, *ε* is the error component which has a logistic random distribution with expected value equal to 0 and variance equal to $\pi /\sqrt{3}$, that accounts for the measurement error. This modelling approach provides a relevant methodology for capturing the sources of influence (independent variables) that explain an ordinal variable (dependent variable) taking into account the measurement uncertainty of such data^17^.

Since ${y}^{\ast }$ is not a deterministic quantity, it is only possible to define the probability to belong to each class:

6 $$\begin{array}{cl}P(y=j|\bar{x}) & =P({\mu }_{j-1} < {\bar{y}}^{\ast }\le {\mu }_{j})\\  & =\,L({\mu }_{j}-\beta \bar{x})-L({\mu }_{j-1}-\beta \bar{x}),\,j=0\div {m}\end{array}$$

where *L* is the logistic cumulative distribution function.

In our work, the ordinal variable (*y*) is the rating class (from 0 to 4) and the independent variable (*x*) is the number of PVS.

### Model Calibration

**Figure 3:**
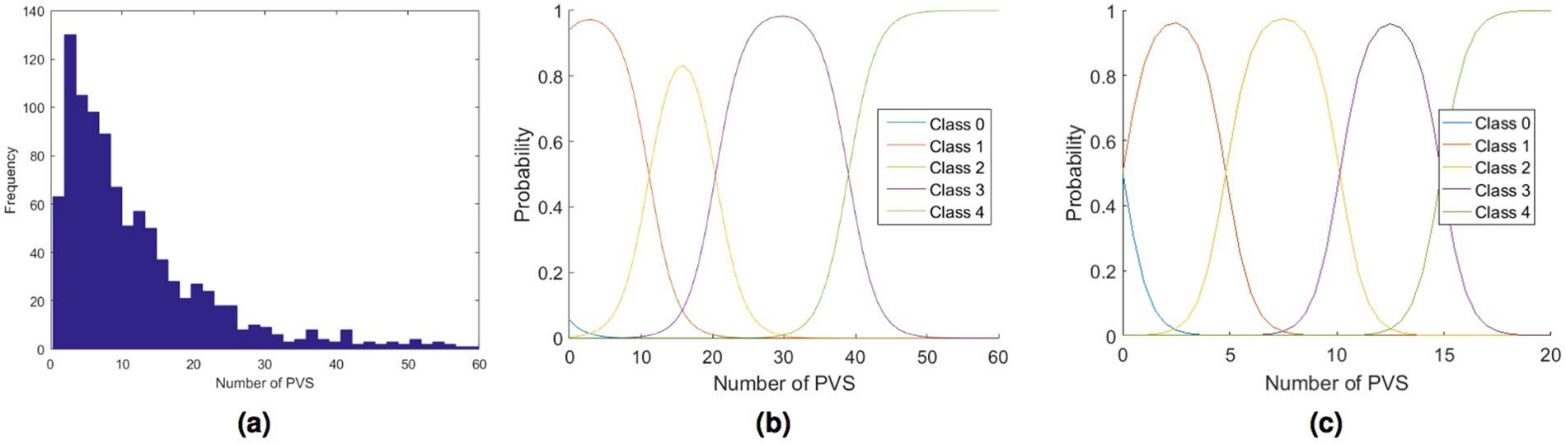
PVS distribution (**a**) of the synthetic dataset generated to calibrate the ordered logit model. Estimated ordered logit model for the Wardlaw (**b**) and the Patankar (**c**) rating scale.

The ordered logit model has been calibrated by maximizing a likelihood function based on a synthetic dataset generated in 3 steps. In the first step 1000 numbers of PVS Count (*PC*_*i*_, *i* = 1, ..., 1000) have been generated using a log-normal distribution (see Fig. 3a), that reflects the observed PVS distribution in known datasets^11^. In the second step, the uncertainty has been simulated for each *PC*_*i*_ casting a New value of PVS Count (*NPC*_*i*_) using a normal distribution with mean equal to *PC*_*i*_ and standard deviation equal to one. Therefore, the probability that *NPC*_*i*_ is included between *PC*_*i*_ − 3 and *PC*_*i*_ + 3 is 0.997. These values reflect our measurements uncertainty^11^. In the third step, a Rating Class (*RC*_*ij*_) has been assigned to each generated *NPC*_*i*_.

Assuming *m* classes, the log-likelihood function can be written as:

7 $$LogL(\mu ,\beta )=\sum _{i\mathrm{=1}}^{1000}\sum _{j\mathrm{=1}}^{m}P(y=j|NP{C}_{i})R{C}_{ij}$$

where *RC*_*ij*_ is equal to one if the *i*^*th*^ generated number belong to the *j*^*th*^ rating class and it is equal to zero otherwise. The Broyden-Fletcher-Goldfarb-Shanno (BFGS) algorithm has been used to estimate the ordered logit parameters.

For the Wardlaw scale^7^, a rating class from 0 to 4, being 0(*none*), 1(1–10), 2(11–20), 3(21–40), 4(>40) PVS, has been assigned to each generated number. The estimated parameters are *β* = 0.514, *μ*_0_ = −2.840, *μ*_1_ = 5.708, *μ*_2_ = 10.497, *μ*_3_ = 20.040, and the model is illustrated in Fig. 3b.

For the Patankar scale^10^ a rating class from 0 to 4, being 0(*none*), 1(1–5), 2(6–10), 3(11–15), 4(>15) PVS, has been assigned to each generated number. The estimated parameters for the Patankar rating scale are *β* = 1.906, *μ*_0_ = 2.269, *μ*_1_ = 9.569, *μ*_2_ = 18.995, *μ*_3_ = 28.639, and the model is illustrated in Fig. 3c.

### Image Preprocessing

Images were preprocessed to generate the Region-of-Interest (ROI) masks. A fuzzy C-means clustering algorithm was applied to T1 images^22^. This is an unsupervised iterative clustering technique that effectively assigns each voxel to one of 4 membership classes: background, Cerebrospinal Fluid (CSF), Gray Matter (GM), and White Matter (WM). After a series of morphological and thresholding operations, the CSF and GM re-labelled voxels were combined to generate the final CSFGM mask which was used for false positive minimization. To avoid PVS mislabelled as GM, an hole filling procedure was used. The Centrum Semiovale (CS) was automatically identified as the region of WM, superior to the lateral ventricles previously obtained using Lesion Explorer^20^. In this paper we focused on the CS rather than the Basal Ganglia (BG), due to the availability of these ROI masks.

### Parameter Optimization

In order to apply the 3D Frangi filtering, the coregistered MRI volumes were first resliced to make 1 *mm* isotropic voxels using linear interpolation. Then volumes have been filtered according to Eqs (2) and (3) and voxels having F(v) larger than a threshold *t* were kept. The two segmentations from T1W and T2W modalities were combined using an AND operation. PVS were identified as the tubular structures with lengths between 3 and 50 *mm*^3,11^, using 3D connected component analysis with 18-neighbourhood rule. This provided the initial PVS binary masks. For each slice we calculated the PVS density as the area of the PVS mask divided by the area of the CS mask. We automatically selected the slice in the CS with highest density of PVS. This slice corresponded to the representative slice having the highest number of PVS selected by the radiologist for assessing the Wardlaw visual ratings^7^. The count of PVS in this slice was derived automatically with 2D connected component labelling. Similarly, the total number of PVS in the entire CS was obtained with 3D connected component labelling. This count of PVS corresponded to the count performed by the radiologist for the Patankar ratings^21^.

A log-likelihood function has been defined to optimize the segmentation parameters: Frangi filter scales *s*_*min*_, *s*_*max*_ and threshold *t*. In this work, we used the default configuration for the other Frangi filter parameters (*α* = 0.5, *β* = 0.5, *c* = 500), as in our previous work^23^ we noted that optimizing these parameters produced essentially similar results, at the cost of a much higher computational time.

Based on the count of PVS (*x*_*i*_(*s*_*min*_, *s*_*max*_, *t*)) for each case *i* we obtained the probabilities of each case *i* to belong to the five rating classes (*P*(*y* = *j*|*x*_*i*_), *j* = 0, …, 4) using the ordered logit model. The PVS visual rating category provided by an expert radiologist was then used to select a probability for each *i* case (${\bar{P}}_{i}$). The sum of the logarithms of these selected probabilities is the log-likelihood function to maximize:

8 $$LogL({s}_{min},{s}_{max},t)=\sum _{i\mathrm{=1}}^{N}log({\bar{P}}_{i})$$

where N is the number of cases.

### Model Validation

Segmentation procedures are commonly evaluated by assessing the voxel-wise spatial agreement between two binary masks, one obtained by the automatic method and a manual one. In our case, the manual segmentation of PVS was not available, as it would have been a very tedious and time consuming task to manually annotate these tiny structures in a reasonable size dataset. Therefore the true number of PVS was also not available. Quantitative comparison with other methods^9,11^ was unfeasible as they have been applied to MR images having different resolution, acquired using different protocols in different cohorts.

The performance of the models was therefore evaluated comparing single-slice PVS automatic counting on segmented images vs validated visual ratings using Spearman’s *ρ* (statistical analysis were performed using MATLAB Robust correlation toolbox^24^). Correspondence of PVS total count and volume vs. visual ratings was also assessed to test generalizability.

## Experiments and Results

For developing and optimizing the segmentation approach, the imaging datasets of 20 representative subjects were selected from a sample of the Sunnybrook Dementia Study (SDS)^10^. These 20 subjects had visual ratings assessed by three raters as summarized in Table 2.

**Table 2:** Ratings available for the Sunnybrook Dementia Study (SDS): ◯optimization scale/rater, ✓ validation scale/rater.

	rater 1	rater 2	rater 3	
modified Wardlaw	◯	✓		exp 1
modified Patankar	◯	✓		exp 2
original Wardlaw			◯	exp 3
original Patankar			✓	exp 3

The optimization procedure has been applied to T1W and T2W MRI sequences. Frangi filter scales (*s*_*min*_ and *s*_*max*_) and two thresholds (*t*_1_ and *t*_2_, one for each modality) have been simultaneously optimized. The 2 binary masks obtained were combined using an AND operation. The range of the parameters that undergo the optimization process has been defined as in Table 3.

**Table 3:** Range of the segmentation parameters to optimize.

	*s*_*min*_	*s*_*max*_	*t*_1_	*t*_2_
min value	0.2	2.0	0.90	0.05
max value	2.0	4.0	0.99	0.50
increment	0.2	0.2	0.01	0.05

The high computational time needed to simultaneously optimize multiple parameters is a common drawback of optimization processes. Indeed, each log-likelihood function evaluation implies filtering all the training samples using Eq. (2), which may become critical in this 3D case. To keep a reasonable computational time, in this research contribution, we limited the search space to a subsets of parameters and roamed through this space using a systematic grid search.

Three sets of experiments were performed as indicated in Table 2. The symbol ◯indicates the scale/rater used for optimization, while ✓ specifies those used for validation.

**Figure 4:**
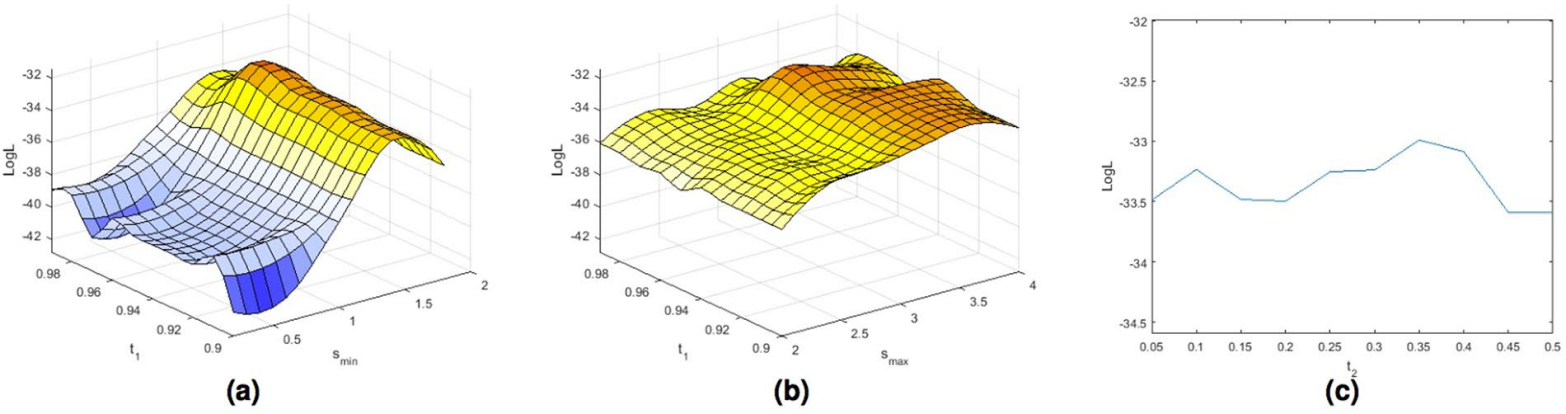
Plots of the log-likelihood function (LogL) obtained using the ordered logit model shown in Fig. 3b estimated with the Wardlaw ratings for a range of examined *s*_*min*_ (**a**) and *s*_*max*_ (**b**) scales and thresholds *t*_1_ values, and threshold *t*_2_ (**c**) values with the best combination of *s*_*min*_, *s*_*max*_ and *t*_1_.

**Figure 5:**
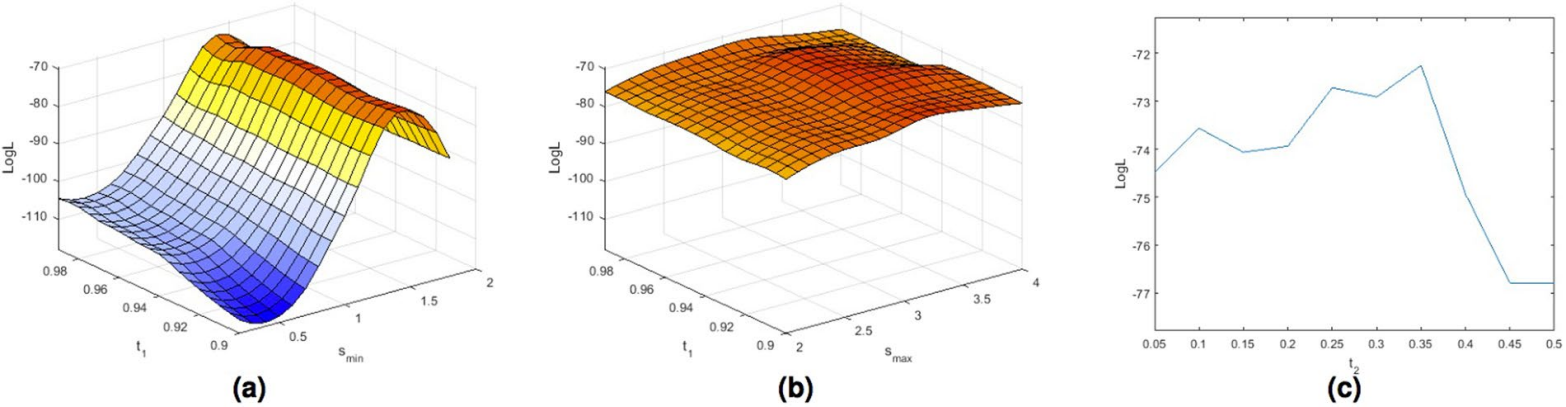
Plots of the log-likelihood function (LogL) obtained using the ordered logit model shown in Fig. 3c estimated with the Patankar ratings for a range of examined *s*_*min*_ (**a**) and *s*_*max*_ (**b**) scales and thresholds *t*_1_ values, and threshold *t*_2_ (**c**) values with the best combination of *s*_*min*_, *s*_*max*_ and *t*_1_.

For illustration Fig. 4a and b show the surface plots for the parameter optimization using the modified Wardlaw rating scale for a range of examined *s*_*min*_ and *s*_*max*_ scales and *t*_1_ values. Figure 4c show the trend of the log-likelihood function (LogL) for a range of examined threshold *t*_2_ values with the the best combination of *s*_*min*_, *s*_*max*_ and *t*_1_. Figure 5 show the surface plots for the parameter optimization using the modified Patankar rating scale. The optimal parameters obtained with the 2 models are very similar (*s*_*min*_ = 1.4, *s*_*max*_ = 3.2, *t*_1_ = 0.96, *t*_2_ = 0.35 for the first model, *s*_*min*_ = 1.4, *s*_*max*_ = 3.2, *t*_1_ = 0.95, *t*_2_ = 0.35 for the second one). From the plots we can observe that the most significant parameter of the Frangi filter is the minimum scale (*s*_*min*_). From these plots it is also clear that the Frangi filter was needed. Indeed for any combination of *s*_*min*_ and *s*_*max*_ the threshold values play a smaller role.

**Figure 6:**
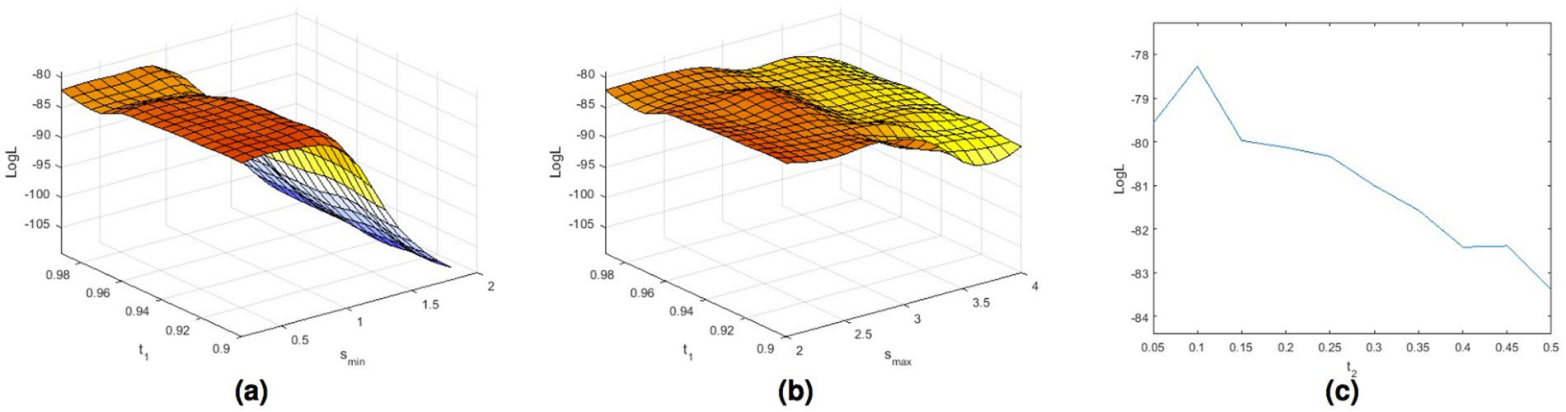
Plots of the log-likelihood function (LogL) obtained using the ordered logit model shown in Fig. 3b estimated with the Wardlaw ratings for a range of examined *s*_*min*_ (**a**) and *s*_*max*_ (**b**) scales and thresholds *t*_1_ values, and threshold *t*_2_ (**c**) values with the best combination of *s*_*min*_, *s*_*max*_ and *t*_1_.

The optimal parameters obtained with the Wardlaw model using PVS assessed by the third rater were slightly different from the previous ones (*s*_*min*_ = 0.2, *s*_*max*_ = 2, *t*_1_ = 0.96, *t*_2_ = 0.1). The plots of the log-likelihood function are shown in Fig. 6. The trend of plots confirms the validity of the model demonstrating that the model was able to adapt to the rater, and finds the best parameters to segment the PVS accounted by that rater.

### Qualitative Evaluation

**Figure 7:**
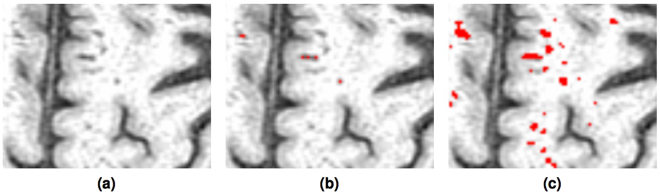
Visual comparison of the PVS segmentation overlaid on T1 (**a**) using the conservative threshold based^10^ method (**b**) and the proposed Frangi filtered (**c**) method.

Magnified views of PVS segmentation using the threshold-based method previously described^10^ and the proposed method are shown in Fig. 7. It is clear that the proposed method detected most of the PVS, including the tiny ones, thanks to the enhancement of tubular structure performed by the Frangi filtering using the appropriate scale. The threshold based method missed them, as it was forced to be conservative in order to distinguish PVS from confounding tissue boundaries.

**Figure 8:**
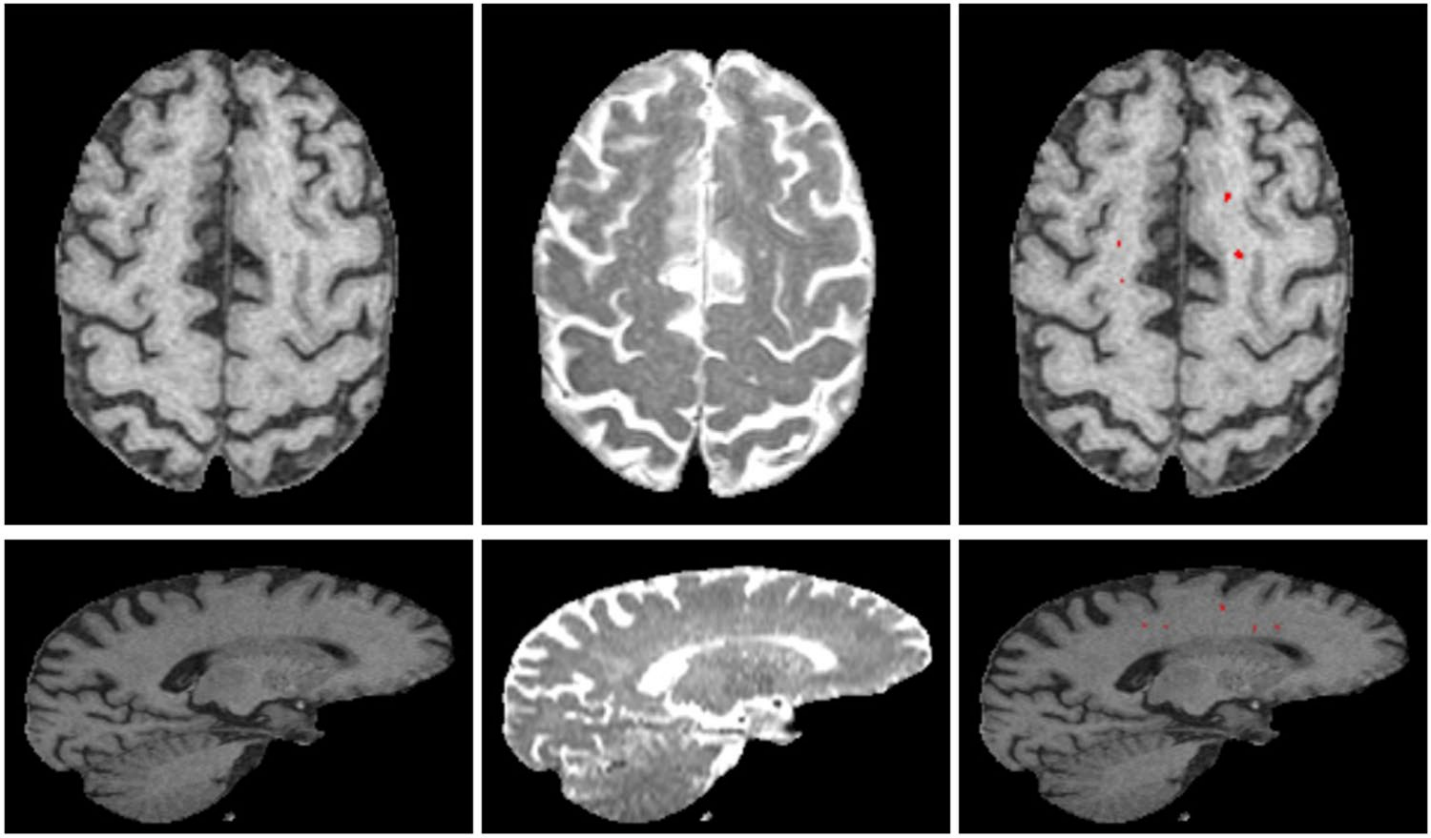
Examples of the final PVS segmentation a case of SDS dataset having few PVS. Axial (top row) and sagittal (bottom) slice of T1W, T2W and PVS overlay (red) on T1W. For illustration, T1W is shown in its native space (256 × 256 × 124) and T2W is shown registered to T1W.

**Figure 9:**
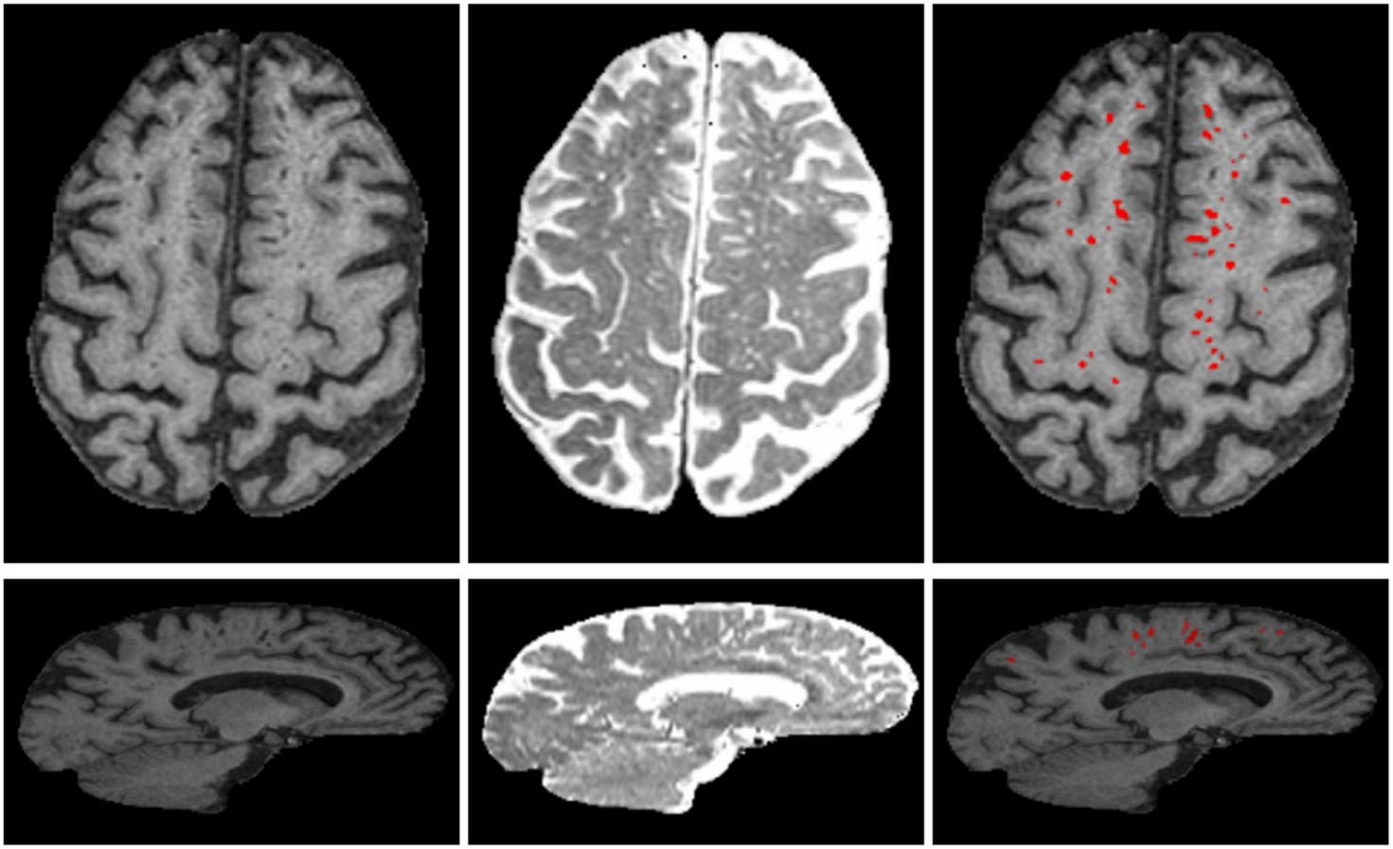
Examples of the final PVS segmentation for a case of SDS dataset having many PVS. Axial (top row) and sagittal (bottom) slice of T1W, T2W and PVS overlay (red) on T1W.

**Figure 10:**
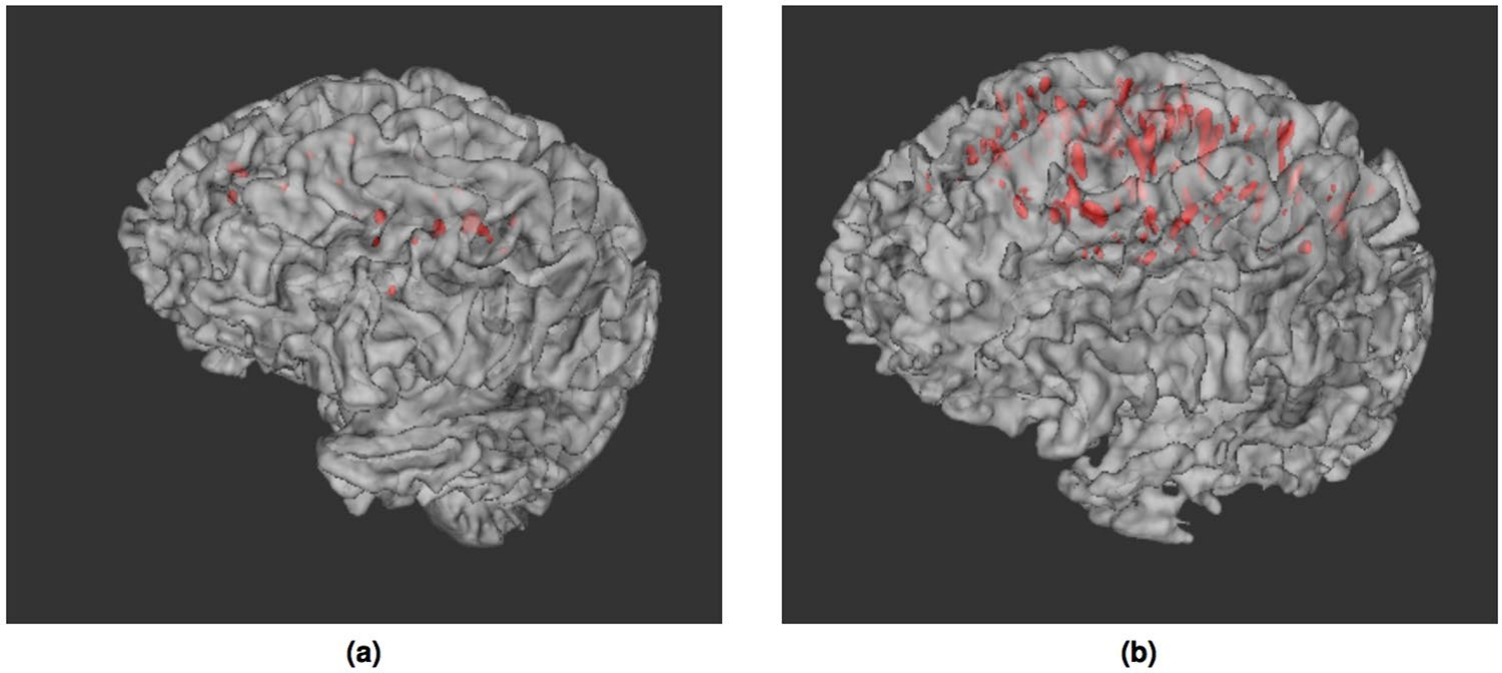
Volume rendering of segmented PVS (red) for two SDS cases having few (**a**) and many (**b**) PVS. PVS volumes overlayed onto a surface render of the brain.

Examples of segmented PVS for two representative SDS cases having few and many PVS are shown in Figs 8 and 9. For each case, we show T1W, T2W and the PVS overlay in red. Volume rendering of the segmented PVS for two cases having few and many PVS are shown in Fig. 10 for visual qualitative evaluation.

### Quantitative Evaluation

When comparing single-slice PVS count obtained from segmented images with the modified Wardlaw and Patankar visual ratings of the second rater, a fair correlation was found for both methods (Spearman’s *ρ* = 0.58, *p* = 0.006 and *ρ* = 0.71, *p* = 0.0004 respectively). However, low and no significant correlation was found with total PVS number in volume in Centrum Semiovale, suggesting low generalizability. This replicates our previous analysis^10^.

For the segmentation results obtained with the optimal parameters of the model optimized with the original Wardlaw scale a stronger correlation between single-slice PVS count vs visual ratings was found (Spearman’s *ρ* = 0.74, *p* = 0.0002). In addition PVS total count and volume correlates with visual rating scores (Spearman’s *ρ* = 0.67, *p* = 0.001 and *ρ* = 0.53, *p* = 0.015, respectively).

### Application to alternative acquisitions

To validate the new PVS segmentation method we applied it to MRI of cases of the Mild Stroke Study (MSS). Visual ratings using the Wardlaw rating^7^ were available for all the cases^4^.

Automatic brain, cerebrospinal fluid (CSF) and normal-appearing white matter extraction were performed on T1W MRI using optiBET^25^ and FSL-FAST^26^ respectively. All subcortical structures were segmented, also automatically, using other tools from the FMRIB Software Library (FSL) and an age-relevant template as per the pipeline described elsewhere^18^. After identifying the lateral ventricles as the CSF-filled structures with boundaries with the subcortical structures, the CS was identified as the region of normal-appearing white matter, superior to the lateral ventricles, present in each of the cerebral hemispheres under the cerebral cortex. T1W sequence and CS region were linearly registered to the T2W-cube images^27^. This preprocessing differs from the one used for the SDS dataset due to the pipelines available at the two research groups^10,18^. The optimization procedure has been applied to T2-cube MRI sequences of 20 patients, and tested on 48 patients of the same study. The optimal parameters obtained for this dataset (*s*_*min*_ = 0.4, *s*_*max*_ = 3.6, *t*_2_ = 0.4) were different from those for the SDS dataset. This confirm the method was able to adapt the parameters to the different voxel-size.

**Figure 11:**
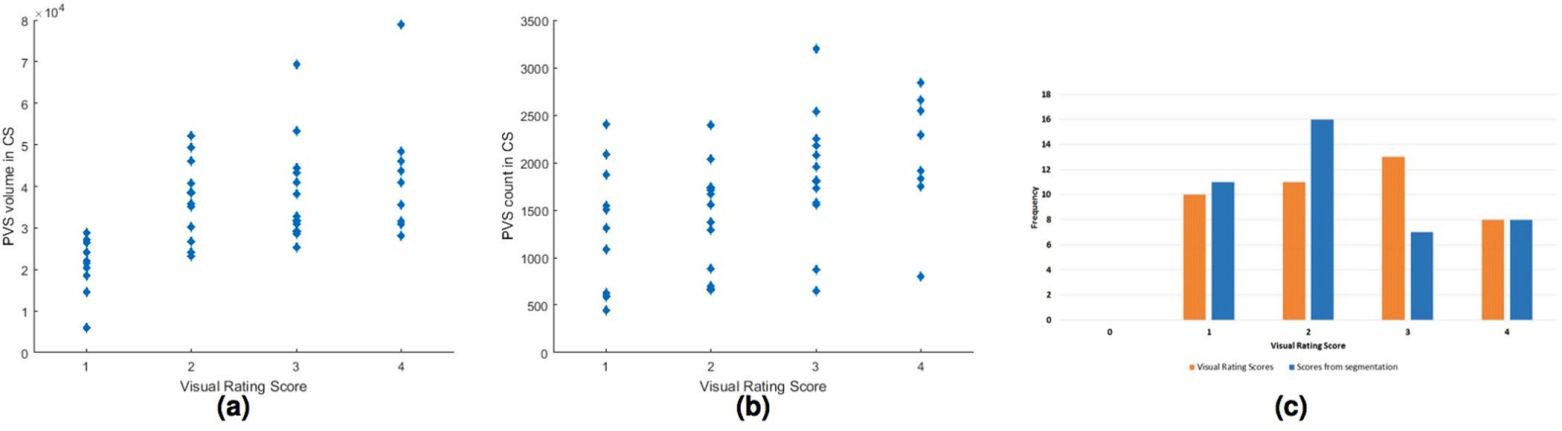
Associations between PVS computational total volume (**a**) and count (**b**) vs. PVS visual rating scores in centrum semiovale (CS) region for the 48 test cases of the MSS dataset. Comparison of PVS computational count condensed into a score of similar range to the visual rating categories (**c**).

PVS total count and volume correlates with visual rating scores (Spearman’s *ρ* = 0.47, *p* < 0.001 and *ρ* = 0.57, *p* < 0.001, respectively). Scatter plots of these associations are shown in Fig. 11a and b. Condensed raw PVS computational count into the same categories of the visual rating scale has a similar distribution of the visual rating scores, as shown in Fig. 11c.

The results of this experiment suggest fair generalizability of the output of the segmentation method vs validated visual rating scores.

## Discussion

The 3D Frangi filter enhances and captures the 3D geometrical shape of PVS, thus this method shows promise for identifying and quantifying PVS that run both longitudinally and transversally in the Centrum Semiovale, avoiding the double-counting limitations of slice-based methods. Centrum Semiovale is more difficult to rate visually than Basal Ganglia, so future application of this method to Basal Ganglia may be more straightforward. The ordered logit model could deal with the measurement uncertainty and the unequal class intervals of the rating scores.

One limitation of this method is that it relies on the image preprocessing step for the ROI masks. If the masks provided by this step are not accurate, the method can detect as PVS boundary of grey matter and gyri. Another limitation of this method is that it requires high resolution and quasi isotropic structural MRI. Very noisy images have been excluded for this study, otherwise any noise spot of tubular shape can be wrongly segmented as PVS. This can be overcome by a learning method. However, learning methods require GT, and not just visual ratings assessment.

The method is fully automatic and therefore free from inter- and intra-rater variability. However, much more testing is required in a wider range of subjects including those with high burden of other ageing and neuroinflammation features. Visual checking and editing is likely to be needed in complex cases, but this remains to be defined.

The quantitative assessment of PVS volume and count is more suitable for longitudinal studies than visual ratings, that tend to be susceptible to ceiling/flooring effects. The accurate segmentation of PVS will allow the analysis of their spatial distribution, orientation and density. The resulting PVS masks could be used, in combinations with other quantitative sequences, to assess other tissue characteristics in adjacent tissue. Moreover, this method will enable the study of the spatial and volumetric relationships of PVS with other markers of SVD, e.g. acute lacunar infarcts, white matter hyperintensities, lacunes, and microbleeds. Additionally, this method shows promise for use in longitudinal studies where PVS burden can be assessed in relation to measures of cerebral blood brain barrier permeability, perfusion and cerebrovascular reactivity.

## Conclusions

We presented an automatic method for 3D segmentation of PVS in conventional brain MRI. The novelty of this work is the fact that the ordered logit model allows use of the visual ratings for Frangi filter parameter optimization in absence of alternative computational ground truth. The automatically segmented PVS count and volume agree with visual ratings. Quantitative measurements will better characterize the severity of PVS in ageing people and their associations with dementia, stroke and vascular diseases. This is the first work to propose a multicentre study of PVS segmentation. It shows excellent multi-centre reproducibility.
